# The mechanisms of skeletal muscle atrophy in response to transient knockdown of the vitamin D receptor *in vivo*

**DOI:** 10.1113/JP280652

**Published:** 2020-12-24

**Authors:** Joseph J. Bass, Abid A. Kazi, Colleen S. Deane, Asif Nakhuda, Stephen P. Ashcroft, Matthew S. Brook, Daniel J. Wilkinson, Bethan E. Phillips, Andrew Philp, Janelle Tarum, Fawzi Kadi, Ditte Andersen, Amadeo Muñoz Garcia, Ken Smith, Iain J. Gallagher, Nathaniel J. Szewczyk, Mark E. Cleasby, Philip J Atherton

**Affiliations:** ^1^ MRC/ARUK Centre for Musculoskeletal Ageing Research and National Institute for Health Research (NIHR) Nottingham Biomedical Research Centre (BRC) School of Medicine University of Nottingham Nottingham UK; ^2^ Department of Cellular and Molecular Physiology Pennsylvania State University College of Medicine Hershey PA USA; ^3^ Department of Sport and Health Sciences University of Exeter Exeter UK; ^4^ Living Systems Institute University of Exeter Exeter UK; ^5^ School of Sport, Exercise and Rehabilitation Sciences University of Birmingham Birmingham UK; ^6^ Mitochondrial Metabolism & Ageing Laboratory, Diabetes and Metabolism Division Garvan Institute of Medical Research New South Wales Australia; ^7^ St Vincent's Medical School, UNSW Medicine, UNSW Sydney Australia; ^8^ School of Health Sciences Örebro University Örebro Sweden; ^9^ Molecular Physiology of Diabetes Laboratory Department of Comparative Biomedical Sciences Royal Veterinary College London UK; ^10^ Institute of Metabolism and Systems Research The University of Birmingham Birmingham UK; ^11^ Department of Bioinformatics – BiGCaT NUTRIM School of Nutrition and Metabolism in Translational Research Maastricht University Maastricht The Netherlands; ^12^ Physiology, Exercise and Nutrition Research Group Faculty of Health Sciences and Sport University of Stirling Stirling UK

**Keywords:** atrophy, metabolism, skeletal muscle, vitamin D

## Abstract

**Key points:**

- Reduced vitamin D receptor (VDR) expression prompts skeletal muscle atrophy.
- Atrophy occurs through catabolic processes, namely the induction of autophagy, while anabolism remains unchanged.
- In response to VDR‐knockdown mitochondrial function and related gene‐set expression is impaired.
- *In vitro* VDR knockdown induces myogenic dysregulation occurring through impaired differentiation.
- These results highlight the autonomous role the VDR has within skeletal muscle mass regulation.

**Abstract:**

Vitamin D deficiency is estimated to affect ∼40% of the world's population and has been associated with impaired muscle maintenance. Vitamin D exerts its actions through the vitamin D receptor (VDR), the expression of which was recently confirmed in skeletal muscle, and its down‐regulation is linked to reduced muscle mass and functional decline. To identify potential mechanisms underlying muscle atrophy, we studied the impact of VDR knockdown (KD) on mature skeletal muscle *in vivo*, and myogenic regulation *in vitro* in C2C12 cells. Male Wistar rats underwent *in vivo* electrotransfer (IVE) to knock down the VDR in hind‐limb tibialis anterior (TA) muscle for 10 days. Comprehensive metabolic and physiological analysis was undertaken to define the influence loss of the VDR on muscle fibre composition, protein synthesis, anabolic and catabolic signalling, mitochondrial phenotype and gene expression. Finally, *in vitro* lentiviral transfection was used to induce sustained VDR‐KD in C2C12 cells to analyse myogenic regulation. Muscle VDR‐KD elicited atrophy through a reduction in total protein content, resulting in lower myofibre area. Activation of autophagic processes was observed, with no effect upon muscle protein synthesis or anabolic signalling. Furthermore, RNA‐sequencing analysis identified systematic down‐regulation of multiple mitochondrial respiration‐related protein and genesets. Finally, *in vitro* VDR‐knockdown impaired myogenesis (cell cycling, differentiation and myotube formation). Together, these data indicate a fundamental regulatory role of the VDR in the regulation of myogenesis and muscle mass, whereby it acts to maintain muscle mitochondrial function and limit autophagy.

## Key points

- Reduced vitamin D receptor (VDR) expression prompts skeletal muscle atrophy.
- Atrophy occurs through catabolic processes, namely the induction of autophagy, while anabolism remains unchanged.
- In response to VDR‐knockdown mitochondrial function and related gene‐set expression is impaired.
- *In vitro* VDR knockdown induces myogenic dysregulation occurring through impaired differentiation.
- These results highlight the autonomous role the VDR has within skeletal muscle mass regulation.

## Introduction

Vitamin D deficiency (25‐hydroxyvitamin D <50 nmol/L) is globally widespread, affecting ∼40% of all adults (Forrest & Stuhldreher, 2011). The classical function of vitamin D is to regulate calcium (Ca^2+^) and phosphate (P_i_) homeostasis to maintain bone health and prevent rickets (Arthur W. Ham, 1934), osteomalacia (Bhan *et al*. 2010) and osteoporosis (Rizzoli *et al*. 2008). However, vitamin D deficiency has also been linked to deterioration of wider musculoskeletal health, including a reduction in skeletal muscle mass/function (Dhanwal *et al*. 2013), a higher risk of falls in old age (Bischoff‐Ferrari *et al*. 2004 *b*), and the exacerbation of cachexia (Dev *et al*. 2011) and type 2 diabetes (Pittas *et al*. 2007). Furthermore, chronic vitamin D deficiency in humans is accompanied by signs of myopathy that can be rescued by vitamin D supplementation (Prabhala *et al*. 2000). Similarly, vitamin D supplementation has been shown to enhance muscle function and myofibre cross‐sectional area (CSA) in the elderly (Sato *et al*. 2005; Ceglia *et al*. 2013) and in athletes (Close *et al*. 2013; Wyon *et al*. 2016), although not consistently (*Halfon et al*. 2015). Nonetheless, in summary, there are multi‐faceted links between vitamin D status and musculoskeletal health. This has led to efforts to elucidate mechanistic links between vitamin D and muscle mass and function.

Consistent with a pro‐myogenic role of vitamin D, exogenous vitamin D promoted the differentiation of muscle cells *in vitro* (Okuno *et al*. 2012; Girgis *et al*. 2014a). Vitamin D exerts its genomic functions through the ubiquitously expressed vitamin D receptor (VDR) (Norman, 2008). Studies have confirmed expression of the VDR (Pike, 2014) and CYP27B1 [inactive 25‐hydroxyvitamin D_3_ > active 1α,25‐dihydroxyvitamin D_3_ (1α,25(OH)_2_D_3_)] in fully differentiated skeletal muscle of both rodents and humans (Srikuea *et al*. 2012). Exogenous vitamin D up‐regulates VDR mRNA and vitamin D metabolism in skeletal muscle (van der Meijden *et al*. 2016). Furthermore, the VDR is required for vitamin D‐induced anti‐proliferative, pro‐differentiation effects (Irazoqui *et al*. 2014), while acute VDR‐knockdown (KD) [i.e. small interfering RNA (siRNA)] silencing results in impaired myogenic differentiation (Tanaka *et al*. 2014). However, acute siRNA of the VDR is inadequate for the characterization of the role of VDR throughout proliferation and terminal myogenesis. Nevertheless, the available *in vitro* data are consistent with a role of VDR expression in the regulation of skeletal muscle metabolism, in the absence of the manipulation of vitamin D bioavailability.

*In vivo* down‐regulation of the VDR has been linked to multiple muscle catabolic disease states, while its transgenic deletion also induces muscle pathology. For instance, clinical studies have shown that VDR expression in muscle decreases with age (Bischoff‐Ferrari *et al*. 2004a; M. Scimeca, F. Centofanti, M. Celi, E. Gasbarra, G. Novelli, A. Botta, 2018), as have *in vivo* experiments, in which whole‐body VDR knockout (VDR‐KO) mice have been shown to exhibit reductions in muscle fibre size and grip strength (Endo *et al*. 2003; Girgis *et al*. 2015). Furthermore, aberrant expression of myogenic regulatory factors (myf5 and myogenin) has been shown in VDR‐KO muscle (Endo *et al*. 2003). However, whole‐body VDR‐KO impacts Ca^2+^/Pi homeostasis and bone metabolism, such that mice require a rescue diet to normalize blood mineral ion levels (Amling *et al*. 1999), which are notoriously difficult to control. Thus, developmental dysregulation and hypocalcaemia in VDR‐KO models preclude the identification of a post‐natal role of the VDR in skeletal muscle.

While regulation of skeletal muscle mass and myogenic development by the VDR has been suggested, the mechanisms of this are ill‐defined. Here, we report a comprehensive study into the autonomous role of the VDR in skeletal muscle. First, we demonstrate that a reduction of function (knockdown) of the VDR in a pre‐clinical model *in vivo* results in myofibre atrophy and an associated up‐regulation of autophagy‐related transcriptional and post‐transcriptional pathways, rather than down‐regulation of protein synthesis. Second, we show that sustained *in vitro* VDR‐KD impairs myogenesis (proliferation and myotube development).

## Materials and methods

### Ethical approval

All animal experimental procedures were undertaken and approved by the Royal Veterinary College's Ethics and Welfare committee and were carried out under UK Home Office licence to comply with the Animals (Scientific Procedures) Act 1986.

### Animal handling

Eight‐week‐old male Wistar rats were housed at 22 ± 0.5°C under a 12 h day/12 h night cycle and acclimatized to their new surroundings for 1 week. Animals were provided with water and a standard chow diet *ad libitum* (Special Diet Services, LBS Biotechnology, London, UK) [contained cholecalciferol (D_3_) at 621.7 IU/kg]. Seven rats underwent *in vivo* electrotransfer (IVE), whereby after 10 days rats were fasted overnight and killed by injection of pentobarbitone. Muscles were rapidly dissected thereafter; transverse sections were mounted on cork tiles in optimum cutting temperature (OCT) medium and snap‐frozen in liquid nitrogen‐cooled isopentane. The remaining muscle was snap frozen by freeze‐clamping and stored at −80°C.

### In vivo electrotransfer

IVE procedures were undertaken as previously described (Cleasby *et al*. 2007). Animals were operated upon under surgical depth anaesthesia, induced and maintained using isofluorane (2.5%), and their hind limbs shaved and prepared with ethanol. Tibialis anterior muscles received six spaced intramuscular injections of 50 μl aliquots of lenti shRNA particles prepared in endotoxin‐free sterile saline at 0.5 mg/ml. Each shRNA cassette contained a U6 promoter, the target hairpin and a termination sequence. VDR‐KD groups received VDR shRNA (Origene, Rockville, MD, USA) (Table 1) into the right tibialis muscle and scramble shRNA into the left tibialis muscle. Immediately following this, one 900 V/cm, 100 μs pulse and four 90 V/cm, 100 ms pulses were administered across the distal limb via tweezer‐electrodes attached to an ECM‐830 electroporator (BTX, Holliston, MA, USA). Animals subsequently received a subcutaneous injection of carprofen (50 mg/kg), before recovery and monitoring from anaesthesia.

**Table 1:** shRNA oligonucleotide sequences

Name	Sequence	Source	Identifier
Rat VDR shRNA 1	AATGGAGATTGCCGCATCACCAAGGACAA	Origene	TL709870
Rat VDR shRNA 2	TCACCTCCGATGACCAGATTGTCCTGCTT	Origene	TL709870
Rat VDR shRNA 3	GCTGGTGGAAGCCATTCAGGACCGCCTAT	Origene	TL709870
Rat VDR shRNA 4	TTGTGCTGGAGGTGTTCGGCAATGAGATC	Origene	TL709870
Rat scrambled shRNA	GCACTACCAGAGCTAACTCAGATAGTACT	Origene	TR30021

### Cell culture

C2C12 murine myoblasts (passage 6−10, ECACC, Salisbury, UK) were grown in Dulbecco's‐modified Eagle's medium (DMEM, Invitrogen, Paisley, UK) supplemented with 10% (v/v) heat‐inactivated fetal bovine serum (FBS, Sigma Aldrich, Poole, UK), penicillin (100 U/ml), streptomycin (100 μg/ml), amphotericin B (250 ng/ml), l‐glutamine (2 mM; all Sigma‐Aldrich) at 37°C in a 5% CO_2_ atmosphere (growth media). Myoblasts were seeded in six‐well plates (Nunclon Delta; Thermo Scientific, Loughborough, UK) and grown until ∼95% confluent (2−3 days); differentiation was induced by switching to medium containing 2% FBS (v/v) (differentiation media). Medium was changed every 48 h; however, in experiments in which signalling was assessed, medium was changed 24 h in advance to avoid associated acute perturbations.

### shRNA interference

The lentiviral plasmid used was based on pLKO.1 Clone ID: RMM3981‐201757375 and targeted the (3’ untranslated region) mouse sequence 5′‐TTAAATGTGATTGATCTCAGG‐3′ of the mouse VDR gene; a non‐targeting scrambled (SCR) shRNA sequence was used as a negative control, with the hairpin sequence CCTAAGGTTAAGTCGCCCTCGCTCTAGCGAGGGCGACTTAACCTTAGG (Addgene, Cambridge, MA, USA). Oligonucleotides were obtained from ITDDNA USA and suspended, annealed and cloned into pLKO.1 at the *Eco*RI and *Age*I sites as per the pLKO.1 protocol from Addgene. DH5α cells were transformed with the resultant plasmids for amplification and isolation. HEK293FT cells (Invitrogen, Carlsbad, CA, USA) were grown in DMEM, the 80−85% confluent plates were rinsed once with Opti‐MEM (Invitrogen) and then incubated with Opti‐MEM for 4 h before transfections. psPAX2 and pMD2.G, along with either scramble or pLKO.1 clones targeting mouse VDR (three clones), were added after mixing with Lipofectamine 2000, as per the manufacturer's instructions (Invitrogen). Opti‐MEM was changed after overnight incubation with DMEM containing 10% FBS without antibiotics to allow cells to take up the plasmids and recover. Culture media was collected 36 and 72 h post‐transfection to obtain viral particles. The viral particles present in the supernatant were harvested after 15 min of centrifugation at 1500 *g* to remove cellular debris. The supernatant was further filtered using a 0.45 μm syringe filter. Supernatant containing virus was either stored at −80°C for long‐term storage or at 4°C for immediate use. C2C12 cells at 60% confluence were infected twice overnight with 3 ml of viral supernatant containing 8 μg/ml polybrene in serum‐free, antibiotic‐free DMEM. Fresh DMEM medium containing 10% FBS, antibiotics and 2 μg/ml puromycin (Sigma) were added the next day. Cells that survived under puromycin selection were either harvested as stable clones and stored or studied following differentiation.

### Cell counts and BrdU assay

Myoblasts were seeded in six‐well plates and counted by trypsinization of adherent cells into 1 ml DMEM. Measurements were made 96 h after seeding and each well was counted in quadruplicate using a haemocytometer. DNA synthesis of myoblasts was measured via the incorporation of BrdU in place of thymidine, and detected by a colorimetric ELISA assay (no. 6813, Cell Signalling Technology, Danvers, MA, USA) following the manufacturer's recommendations. Cells were seeded in a 96‐well plate (Nunclon Delta; Thermo Scientific) with the addition of BrdU at a final 1× concentration 24 h before measurement.

### Cell cycle analysis

Myoblasts were seeded in six‐well plates and allowed to proliferate for 24 h before being synchronized to G1/early S phase by incubation with 2 mmol/l hydroxyurea (Sigma‐Aldrich) for 15 h. The cell cycle lock was then released by changing the medium. After 12 h the cells were collected by trypsinization and centrifuged, before being re‐suspended in PBS/1% FBS and fixed in ice cold 80% ethanol for at least 1 h. The cells were pelleted by centrifugation at 300 *g* and the ethanol‐decanted pellet was washed in PBS. The cells were then incubated in propidium iodide (PI) staining solution [0.1% Triton X‐100, 10 μg/ml PI (Sigma‐Aldrich), 100 μg/ml RNase in PBS) for 30 min in the dark, before flow cytometry analysis on a Coulter FC 500 device (Beckman Coulter).

### Myotube morphology of cultured cells

The medium was aspirated from the differentiated cells (Day 6) which were washed twice in 2 ml PBS before fixation in 2 ml PBS and methanol/acetone (2:1:1) for 5 min at room temperature. Fluoroshield mounting medium with DAPI (Sigma Aldrich) was applied to allow visualization of myonuclei. Normal light and fluorescent images were obtained and merged, and the myotube diameters were then measured using ImageJ software (National Institutes of Health, Frederick, MD, USA). Myotubes were defined as cells containing three or more myonuclei and measured at three equidistant points along their length. Five random fields of view were chosen per well from four wells, with five myotubes per view being measured on blinded images. Myotubes were stained with phalloidin (Life Technologies, Manchester, UK) after fixing in a 1:5 dilution of 2% horse serum/PBS before visualization.

### Protein/DNA/RNA measurements

Total cellular alkaline soluble protein, DNA and RNA were analysed spectrophotometrically, as previously described (Crossland *et al*. 2013). For rat muscle samples, frozen whole muscle (∼10 mg) was desiccated under vacuum, before the addition of 0.3 mol/l NaOH and homogenization in a bead beater. For cell culture experiments, cells were scraped into 0.3 mol/l NaOH and incubated at 37°C for 30 min before an aliquot was taken for the measurement of total protein using a Nanodrop (Thermo Scientific). Thereafter, 1 M perchloric acid (PCA) was added to the remaining sample, which was incubated at 4°C for 10 min, before centrifugation at 3000 *g* for 10 min, washing in 0.2 mol/l PCA, and collection of the supernatant for RNA measurement. The resulting pellet was incubated at 70°C for 1 h in 2 M PCA, before centrifugation at 5000 *g*, washing in 2 M PCA, and collection of the supernatant for DNA measurement. RNA and DNA were quantified by measuring the absorbances at 260 and 275 nm, and 268 and 284 nm, respectively.

### Measurement of muscle protein synthesis (MPS)

#### Cells

Measurements of MPS were made in differentiated cells using the stable isotope tracer deuterium oxide (D_2_O) (Crossland *et al*. 2013). The medium was changed 24 h before the addition of D_2_O (70 at.%) to an enrichment of 5%. Cells were incubated for 2 h under normal conditions, then 1 ml of medium was collected and cells were scraped into cold homogenization buffer [50 mM Tris·HCl (pH 7.4), 50 mM NaF, 10 mM β‐glycerophosphate disodium salt, 1 mM EDTA, 1 mM EGTA and 1 mM activated Na_3_VO_4_].

#### Rats

Seven days after IVE, the animals were administered a D_2_O bolus by oral gavage (7. 2 ml/kg, 70 at.%). For basal and maximal D_2_O body water enrichment, two animals were killed prior to and 2 h after oral gavage (respectively) and blood was collected in pre‐chilled tubes containing lithium heparin. These were subsequently cold‐centrifuged at 1750 *g*, and plasma aliquots were frozen at −80°C. Ten days after IVE, the animals were overnight fasted and killed, before blood and muscle was collected.

Samples were prepared as previously described (Wilkinson *et al*. 2014). Briefly, ∼50 mg of muscle was homogenized in ice‐cold homogenization buffer, before continuous vortexing for 10 min, and centrifugation at 13,000 *g* for 5 min at 4°C. For cells, scraped lysates were homogenized on ice, before centrifugation as previously mentioned. Pellets were washed in 70% ethanol, before being hydrolysed overnight at 110°C in 1 ml of 0.1 mol/l HCl and 1 ml of H^+^ dowex resin.

The hydrolysed amino acids were eluted into 2 mol/l NH_4_OH, then evaporated to dryness. The deuterium labelling of protein‐bound alanine was determined though conversion to its tert‐butyldimethysilyl derivative and assessed by single ion monitoring (SIM) of *m*/*z* 260 and 261 by gas chromatography‐mass spectrometry. D_2_O precursor enrichment of both plasma and cell culture medium was measured using a modified acetone exchange method (Yang *et al*. 1998) that was previously used to assess MPS in cell culture (Crossland *et al*. 2013). Two microlitres of 10 M NaOH and 1 μl acetone were added to 100 μl of sample medium and vortex‐mixed for 15 s, before incubation at room temperature for 24 h, allowing the exchange of hydrogen atoms for deuterium. Acetone was extracted into n‐heptane and injected into a gas chromatograph‐mass spectrophotometer, and D_2_O enrichment measured via SIM of *m*/*z* 58 and 59. For rats, basal plasma samples were used to determine the natural D_2_O enrichment, with maximal enrichment occurring 2 h after D_2_O ingestion and the final plasma sample used to average temporal precursor enrichment . All samples were referenced to a standard curve of known D_2_O enrichments, and fractional synthetic rate (FSR) was calculated using the following equation:

$$\mathrm{FSR}\left(\%/h\right)=\left[\left(\mathrm{MP}E_{\mathrm{Ala}}\right)\right]/\left[3.7\times \left(\mathrm{MP}E_{\mathrm{MW}}\right)\times t\right]\times 100$$

where MPE_Ala_ represents protein bound alanine enrichment, MPE_MW_ represents precursor enrichment and *t* signifies time in hours (Gasier *et al*. 2010); 3.7 represents the average number of deuteriums labelled in the precursor alanine.

### qRT‐PCR

RNA from cultured cells and skeletal muscle was extracted in TRizol reagent (Invitrogen 15596026) and reverse transcribed using a High‐Capacity cDNA Reverse Transcription kit (Applied Biosystems 4368814). Quantitative reverse transcriptase PCR (qRT‐PCR) was performed on a ViiA7 Real‐Time PCR system (Life Technologies) with SYBR Select Master Mix (Applied Biosystems 4472908) and primers designed in‐house using Primer Express (Table 2). Quantification was performed using the 2^−ΔΔCT^ method and normalized to GAPDH.

**Table 2:** qRT‐PCR primers

**Gene**	**Accession number**		**Primer sequence**
**Mouse**			
GAPDH	NM_008084.3	F	GGGAGCCAAAAGGGTCATCA
		R	TGATGGCATGGACTGTGGTC
VDR	NM_009504.4	F	GCTATTCTCCAAGGCCCACA
		R	CCGGTTCCATCATGTCCAGT
**Rat**			
GAPDH	NM_017008.4	F	ATCCCGCTAACATCAAATGG
		R	GTGGTTCACACCCATCACAA
VDR	NM_017058.1	F	GGTTTCTTCAGGCGGAGCAT
		R	GGTGATGCGGCAATCTCCAT
Trim63 (Murf1)	NM_080903.1	F	CACCTTCCTCTTGAGTGCCA
		R	CTCAAGGCCTCTGCTATGTGT
Fbxo32 (Atrogin‐1)	NM_133521.1	F	AGCTTGTGCGATGTTACCCA
		R	GGTGAAAGTGAGACGGAGCA
Fbxo40	XM_006248404.3	F	CGGGGTTGGCATAAGTGCTA
		R	CAGAGGACCCGAGTTGACTTC
PSMD11	NM_001107027.1	F	CGACCCAATCATCAGCACAC
		R	GGCCTCTTACCAGACAGACAG
ATG5	NM_001014250.1	F	CAGAAGCTGTTCCGTCCTGT
		R	CCGTGAATCATCACCTGGCT
ATG7	NM_001012097.1	F	CAGCCTGTTCATCCAAAGTTCTTG
		R	CTGTGGTTGCTCAGACGGT
Ctsl	NM_013156.2	F	CTATCGCCACCAGAAGCACA
		R	ACCACACTGGCCCTGATTCT
Casp3	NM_012922.2	F	CGGACCTGTGGACCTGAAAA
		R	CGGCCTCCACTGGTATCTTC
Capn2	NM_017116.2	F	TCGGCATCTATGAGGTCCCA
		R	ATTCTTGTGGGGCTCGAAGG

### Western blotting

To quantify signalling molecules, rat muscles were homogenized in ice‐cold homogenization buffer using clean sharp scissors. Samples were centrifuged at 11,000 *g* for 10 min at 4°C, and the supernatant was removed and quantified by a Nanodrop. Extraction of rat VDR proteins required homogenization and preparation in a hyperosmolar lysis buffer (HLB) [urea 6.7 M, glycerol 10%, Tris‐HCl 10 mM, SDS 1%, DTT 1 mM, PMSF 1 mM, and Protease Inhibitor Cocktail tablet (Roche, West Sussex, UK)], as previously described (Girgis *et al*. 2014b). Cell lysates were homogenized and centrifuged as previously stated, before protein content was quantified by a Nanodrop. All samples were diluted in homogenization buffer and Laemmli loading buffer to the same concentration.

Samples were loaded onto Criterion XT Bis‐Tris 12% SDS‐PAGE gels (Bio‐Rad, Hemel Hempstead, UK) for electrophoresis for 1 h at 200 V. Separated proteins were transferred onto a PVDF membrane for 45 min at 100 V, then blocked in 5% low‐fat milk in Tris‐buffered saline and 0.1% Tween‐20 (TBST) for 1 h at room temperature. Membranes were then incubated at 4°C overnight in 5% milk in TBST primary antibody solutions. Afterwards, membranes were washed three times for 5 min with TBST and incubated for 1 h at room temperature in their respective HRP‐conjugated secondary antibody, anti‐rabbit (Cell Signalling Technologies) 1:2000 5% low‐fat milk in TBST, anti‐mouse (Cell Signalling Technologies) or 1:2000 5% low‐fat milk in TBST. Membranes were washed three times for 5 min in TBST, incubated for 5 min in enhanced chemiluminescence reagent (Millipore, Watford, UK) and visualized using a Chemidoc XRS. Bands were quantified using ImageLab software and normalized to total loaded protein visualized by Coomassie brilliant blue or Ponceau staining (Bass *et al*. 2017).

Primary antibodies against p‐AKT Ser473 (1:2000, #4060), Pan‐AKT (1:2000, #4685), p‐TSC2 Thr1462 (1:2000, #3611), TSC2 (1:2000, #4308), p‐mTOR Ser2448 (1:2000, #2976), mTOR (1:2000, #2972), p‐p70S6K1 Thr389 (1:2000, #9234), p70S6K1 (1:2000, #2708), p‐S6RP Ser235/236 (1:2000, #2211), S6RP (1:2000, #2217), p‐4e‐BP1 Thr37/46 (1:2000, #2855), 4e‐BP1 (1:2000, #9644), p‐eIF4E Ser209 (1:2000, #9741), eIF4E (1:2000, #9742), Beclin 1 (1:2000, #3495), p‐AMPK Thr172 (1:2000, #2535), p‐Raptor Ser792 (1:2000, #2083) and LC3B (1:1000, #2775) were from Cell Signalling Technologies. Primary antibody against Cathepsin L (1:2000, Ab6314) was from Abcam (Cambridge, MA, USA). Primary antibody against VDR (D‐6) (1:2000, SC‐13133) was from Santa Cruz (Santa Cruz, CA, USA). Mitochondrial complex expression was undertaken as previously described (Ashcroft *et al*. 2020). MitoProfile OXPHOS antibody cocktail (110413) was purchased from Abcam and citrate synthase (SAB2701077) was from Sigma Aldrich.

### Immunofluorescence

Muscle cross‐sections 5 μm thick were cut at −22°C using a Cryostat, before mounting on glass slides and air‐drying at room temperature. Sections were fixed in acetone/ethanol (3:1) for 5 min, and washed three times in PBS. Fibre CSA and VDR expression analysis was undertaken at the University of Birmingham. For CSA analysis, primary antibodies towards VDR (Rabbit, Ab109234, Abcam) and dystrophin [Mouse, MANDYS1(3B7), Developmental Studies Hybridoma Bank, Iowa City, IA, USA] were diluted in 5% goat serum in PBS at 1:50 and 1:200, respectively. Antibody solutions were applied to each section before incubation for 2 h in a humidity chamber at room temperature, then washed in PBS three times. Secondary fluorescent anti‐rabbit (Alexa Fluor 594, A11012, Invitrogen) and anti‐mouse (Alexa Fluor 488, A21121, Invitrogen) antibodies were diluted in PBS 1:200 and sections were incubated for 30 min as before. Slides were then washed three times in PBS, and 1:1000 DAPI stain (Invitrogen) was applied for 5 min before three PBS washes. Mounting medium (Invitrogen) was applied to each section and dried in darkness overnight. Additional sections were probed using anti‐MHC IIa (SC‐71) or anti‐MHC IIb (BF‐F3) diluted 1:50 in PBS. All sections were imaged in a blinded fashion using a Nikon Eclipse E600 and analysed using Image Pro3D capture software. Three random fields of view were measured per section.

### RNA‐sequencing analysis

RNA was extracted from snap‐frozen muscle using an RNeasy mini kit (Qiagen, Valencia, CA, USA), following the manufacturer's recommendations. All RNA samples had RNA integrity number (RIN) (Schroeder *et al*. 2006) scores of greater than 8. RNA was prepared using the Tru‐Seq RNA library preparation kit (Illumina) and RNA‐sequencing (RNA‐seq) was carried out by Edinburgh Genomics using the Illumina HiSeq 4000 platform, which generated 75 bp paired‐end reads. Following quality control and base‐calling, tag data were examined with Fast QC and adaptor sequences were trimmed where necessary using Trimmomatic (Bolger *et al*. 2014; Andrews, 2016). Unpaired reads were found to be of low quality and were dropped from the analysis, with no set of paired reads failing quality control. Alignment and feature counts were created using the Rsubread package in R and the edgeR package to examine differential expression (Robinson *et al*. 2010; Liao *et al*. 2013). The count data were filtered as recommended by the authors of edgeR by identifying the CPM at a count of 10 (Lun *et al*. 2016). Subsequent normalization was with the trimmed mean of M‐values method (Robinson & Oshlack, 2010). Differential expression was analysed using the glmFit function of edgeR with design matrices to take account of biological pairing between treated and control limbs. Subsequent geneset testing was carried out using the GSEABase library (Morgan *et al*. 2017) in R, using genesets from the Molecular Signatures Database, maintained by the Broad Institute (Liberzon *et al*. 2011).

PathVisio v 3.3.0 (van Iersel *et al*. 2008; Kutmon *et al*. 2015) was used to construct all pathway analysis, with pathways from the WikiPathways repository (Kutmon *et al*. 2016). The *Rattus norvegicus* Derby Ensembl 91 database was used for the identity‐mapping of genes, with log fold changes (</>0.26) of each gene mapped to pathway nodes and differently expressed genes were visualized.

### Statistical analysis

The results are displayed as mean ± SD. The primary measure was the assessment of MPS, requiring a sample size of *n* = 7 animals for paired contralateral design (Power = 0.8, *d* = 1.64, α = 0.05). All analysis was performed by an unpaired (or paired where appropriate) *t*‐test for two group comparisons or ANOVA with Bonferroni or Tukey *post hoc* analysis between multiple groups on GraphPad Prism7. A *P* value < 0.05 was considered to represent statistical significance.

## Results

### In vivo VDR‐KD induces skeletal muscle atrophy

**Figure 1:**
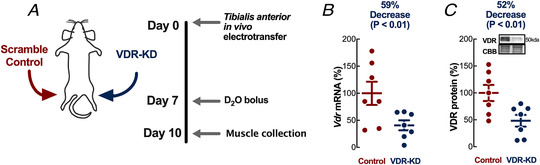
*In vivo* experimental design and grouping *A*, schematic design of *in vivo* experiments. *B*, confirmation of contralateral VDR‐KD by qRT‐PCR (*N* = 7). *C*, representative western blot and quantification of VDR‐KD (*N* = 7). Scale bars represent 200 μm. Data are individual values with mean ± SD. Data were analysed using paired *t*‐tests. [Color figure can be viewed at wileyonlinelibrary.com]

**Figure 2:**
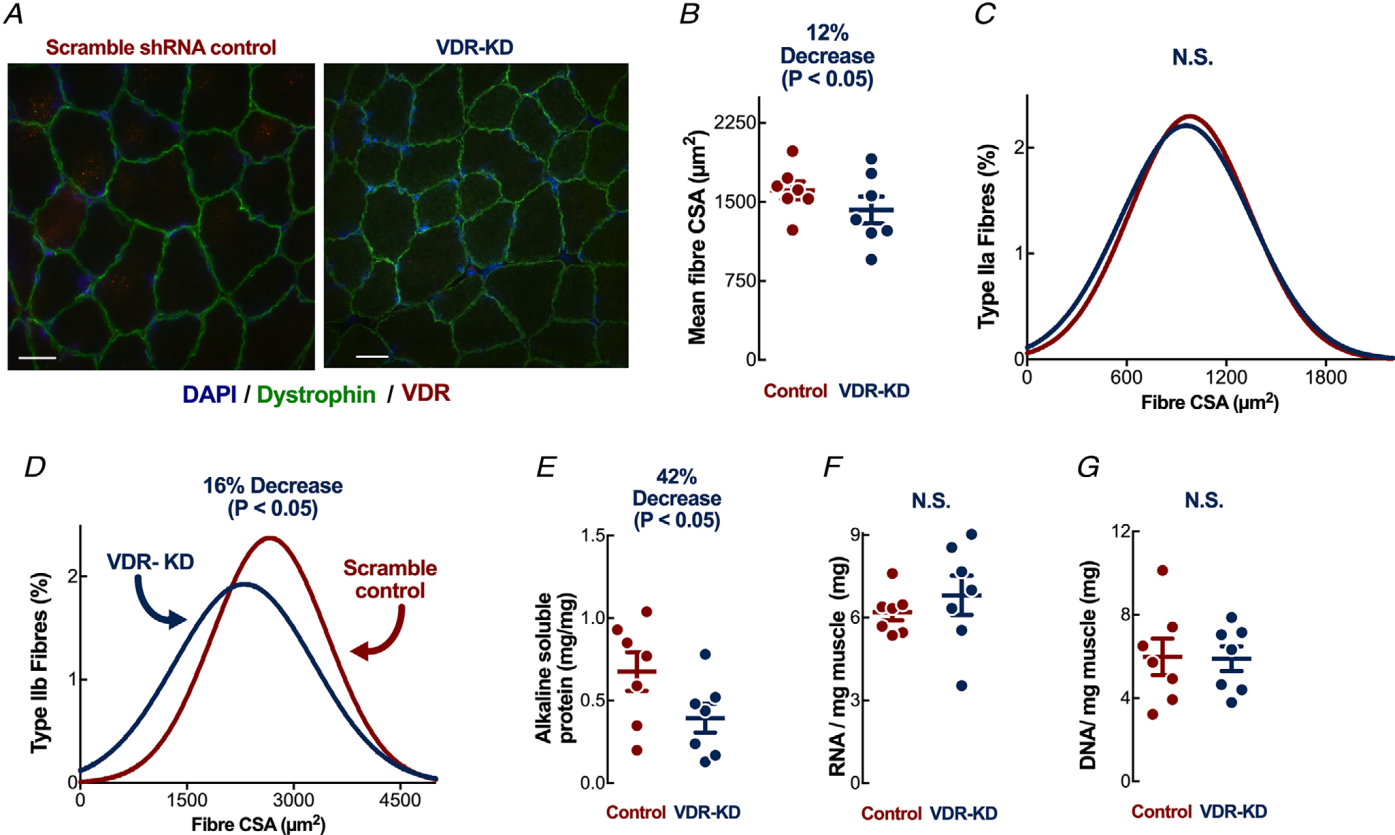
*In vivo* VDR‐KD results in muscle fibre atrophy *A*, representative images of muscle fibres stained for dystrophin (green), DAPI (blue) and VDR (red). Scale bars represent 200 μm. *B*, all fibre CSA analysis; *C*, Type IIa; and *D*, IIb fibre CSA distribution. Three random fields of view were measured per section in both L and R TA muscles in each animal (*N* = 7), with CSA measured for all intact fibres. *E*, alkaline soluble protein measures; *F*, RNA; and *G*, DNA quantification per mg dried muscle (*n* = 7). Data are individual values with mean ± SD. Data were analysed using paired *t*‐tests. [Color figure can be viewed at wileyonlinelibrary.com]

To avoid the confounding effects of VDR‐mediated developmental and hypocalcaemia‐related dysregulation inherent to germline genetic manipulation, we modified VDR expression in post‐natal skeletal muscle. Employing established IVE (Cleasby *et al*. 2005) of vectors expressing *Vdr* shRNA (or scrambled sequences in the contralateral limb, Fig. 1*A*), we confirmed *Vdr* knockdown (VDR‐KD) in rat tibialis anterior (TA) by qRT‐PCR (VDR‐KD: −59 ± 20%) and immunoblotting (VDR‐KD: −52 ± 29%) (Fig. 1*B* and *C*). Functional protein suppression by IVE is persistent and acute inflammation abates after 7 days in skeletal muscle (Cleasby *et al*. 2005). To determine the effects of VDR‐KD on fibre CSA, we immunostained fibres for dystrophin (Fig. 2*A*), and showed that VDR‐KD fibres were smaller (Fig. 2*B*) than contralateral control limb myofibres. Because tibialis muscle mainly consists of fast myosin isoforms, cryosections were immunostained for myosin IIa and IIb, which revealed marked reductions in type IIb fibre CSA following VDR‐KD (Fig. 2*C* and *D*). To verify myofibre atrophy, we also quantified muscle alkaline soluble protein (ASP) content and observed decreases in VDR‐KD muscles (Fig. 2*E*). Analyses of total RNA and DNA content demonstrated no difference between VDR‐KD and control muscle (Fig. 2*F* and *G*).

### Anabolic signalling is unaffected, whereas autophagic processes are upregulated by VDR‐KD

**Figure 3:**
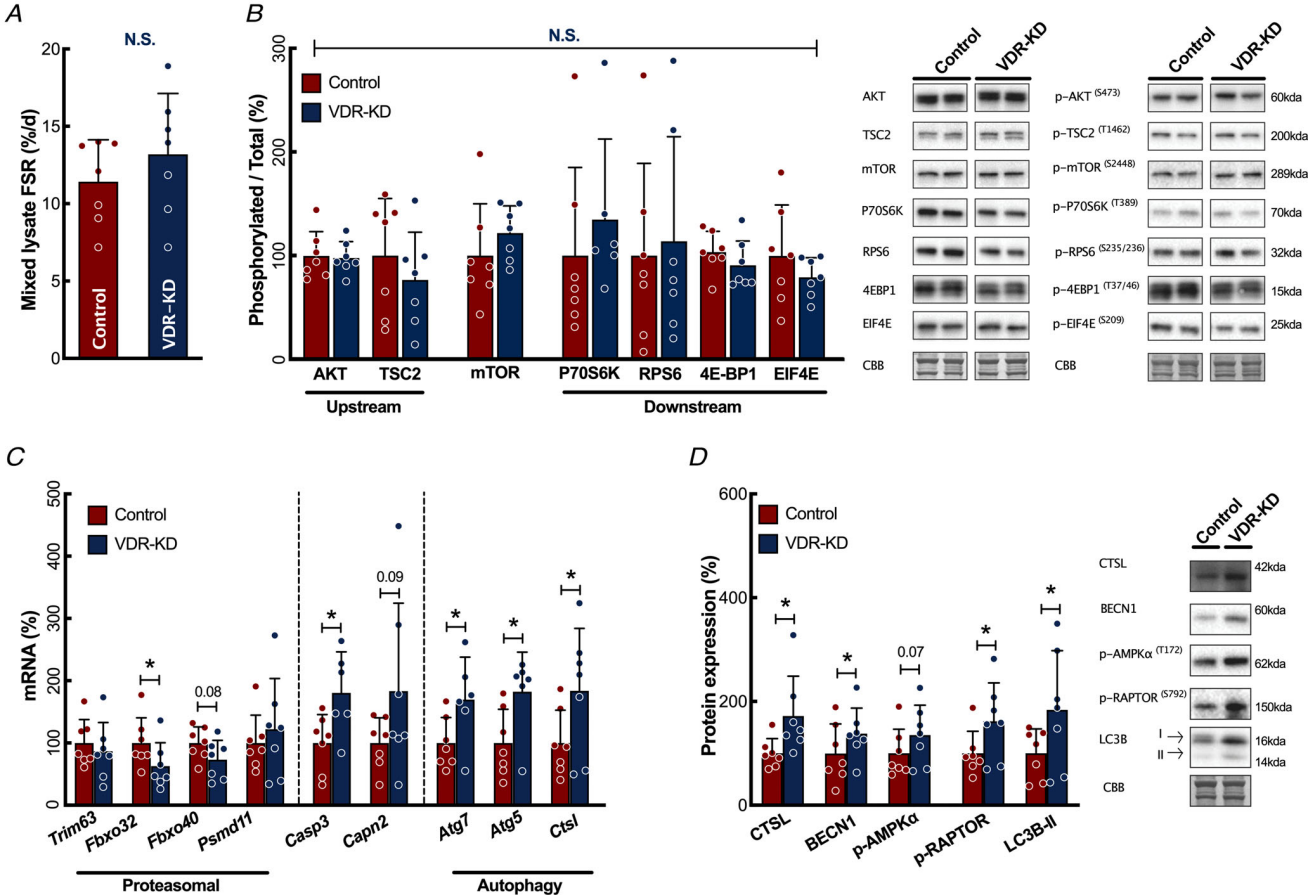
*In vivo* VDR‐KD increases autophagy‐related pathways *A*, measurement of mixed lysate MPS rates by D_2_O incorporation (*N* = 7). *B*, quantification and representative western blots of phosphorylated and total protein anabolic signalling intermediates (*n* = 7). *C*, qRT‐PCR measurement of proteolysis‐related gene expression. *D*, quantification and representative western blots of autophagy‐related protein expression (*n* = 7). Data are individual values with mean ± SD. ^*^ *P* < 0.05, ^**^ *P* < 0.01 between the indicated groups. Data were analysed using paired *t*‐tests. [Color figure can be viewed at wileyonlinelibrary.com]

Because fibre atrophy can result from an impairment in MPS, we quantified MPS using the stable isotope tracer deuterium oxide (D_2_O) and found that global (i.e. across multiple muscle fractions, including myofibrillar, collagenous and sarcoplasmic) MPS rates were not affected by VDR‐KD (VDR‐KD 13.2 ± 4%d *vs*. Control 11.4 ± 2.7%d, Fig. 3*A*). Furthermore, neither protein abundance nor activity (phosphorylation) of indispensable muscle anabolic signalling pathways (AKT/mTORc1) were significantly reduced (e.g. p‐mTOR VDR‐KD 116 ± 78% *vs*. Control 100 ± 67%, Fig. 3*B*). These results suggest that the VDR‐KD‐induced myofibre atrophy and associated net loss of muscle protein were not due to changes in MPS, but instead due to greater proteolysis.

To determine whether fibre atrophy occurred through an alteration in muscle protein breakdown (MPB), we quantified the mRNA abundance of established markers of proteolytic degradation (Sandri, 2013). We identified activation of autophagy markers (e.g. *Atg*5, VDR‐KD 183 ± 64% *vs*. Control 100 ± 54%, *P* = 0.035, *Atg7* VDR‐KD 170 ± 68% *vs*. Control 100 ± 41%, *P* = 0.018) and induction of caspase and calpain pathways (*Casp3* VDR‐KD 181 ± 66% *vs*. Control 100 ± 46%, *P* = 0.044, Fig. 3*C*). In contrast, there were no effects on proteasomal systems (Fig. 3*C*). Additional immunoblotting for validated autophagy markers showed an increase in protein abundance of both lysosomal enzymes and upstream regulatory pathways in VDR‐KD muscles [Cathepsin L (CTSL) VDR‐KD 172 ± 77% *vs*. Control 100 ± 28%, *P* = 0.031), light chain 3B‐II (LC3B‐II; VDR‐KD 183 ± 114% *vs*. Control 100 ± 47%, *P* < 0.045)] and p‐RAPTOR^Ser792^ (VDR‐KD 162 ± 74% *vs*. Control 100 ± 43%, *P* = 0.022) (Fig. 3*D*).

### RNA‐seq analysis demonstrated that VDR‐KD up‐regulates autophagic geneset and down‐regulates mitochondrial metabolic processes

**Figure 4:**
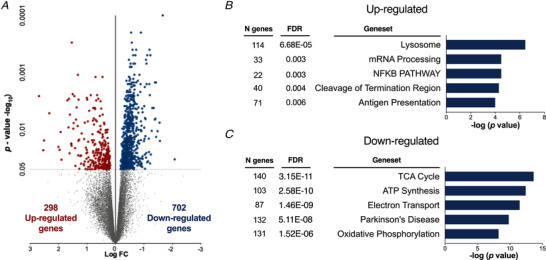
VDR‐KD upregulates autophagy‐related gene‐sets whilst downregulating mitochondrial metabolic processes *A*, volcano plot of *P* < 0.05 statistically significant up‐/downregulated genes. *B*, top five upregulated and downregulated gene‐sets from the Molecular signatures database for VDR‐OE muscles (*n* = 7). See also Supplemental file 1. [Color figure can be viewed at wileyonlinelibrary.com]

**Figure 5:**
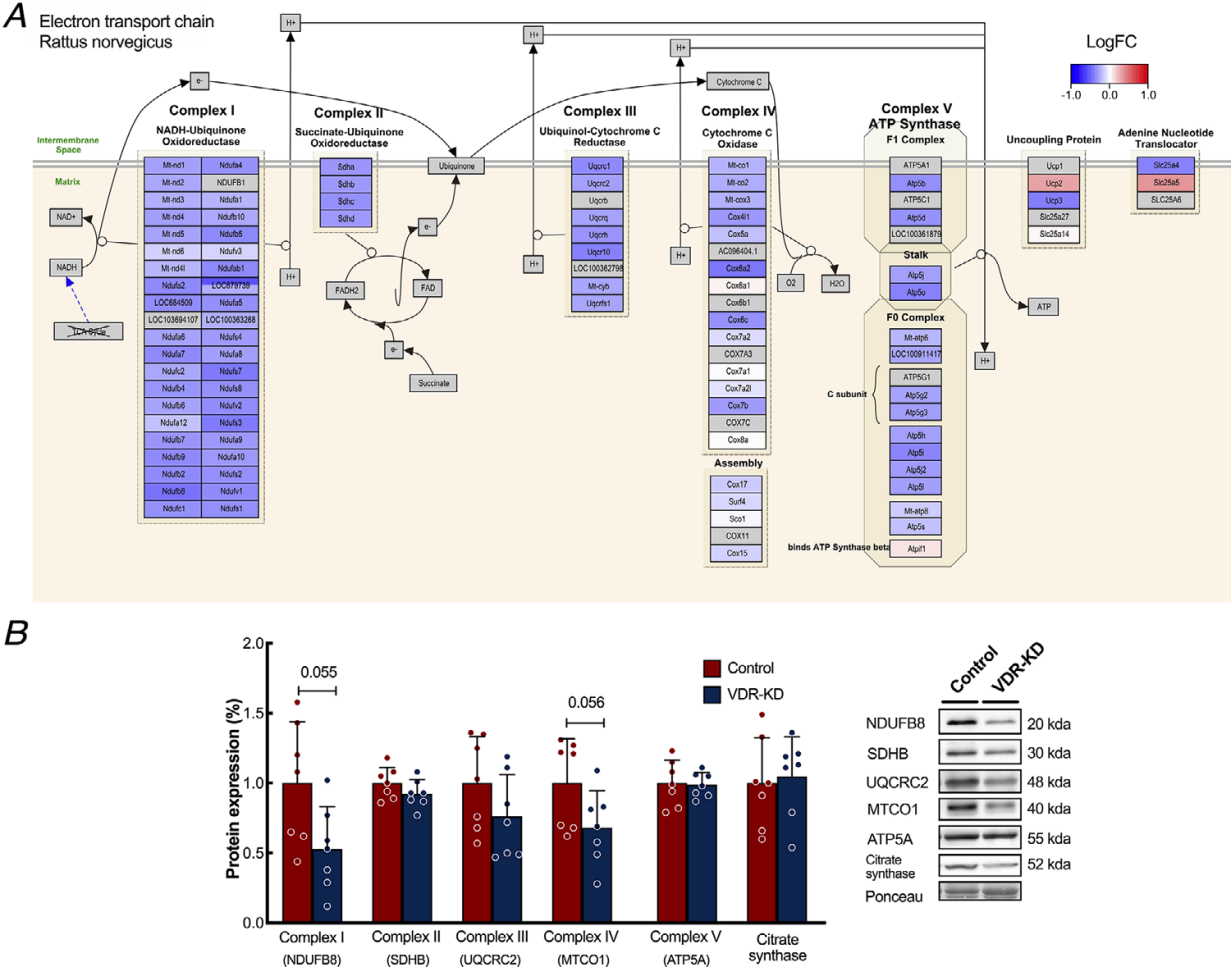
*In vivo* VDR‐KD reduces electron transport chain‐related gene expression *A*, RNA‐seq pathway analysis of *Rattus norvegicus* electron transport chain gene expression. Log fold changes are shown as a gradient from red (upregulated) to blue (downregulated). *B*, quantification and representative western blot analysis of individual mitochondrial electron transport chain complex protein expression (*n* = 7). Data are individual values with mean ± SD. *P* values are for comparisons between the indicated groups. Data were analysed using paired *t*‐tests. [Color figure can be viewed at wileyonlinelibrary.com]

To identify the changes in global regulatory genes associated with the myofibre atrophy induced by VDR‐KD, we utilized RNA‐seq of cDNA libraries generated from VDR‐KD muscles (GSE110507) and contralateral scramble‐transfected controls. VDR‐KD induced differential expression of 1000 genes (*P* < 0.05) (Fig. 4*A*) and multiple genesets [*n* = 107, false discovery rate (FDR) <5%] (Supplemental file 1). Notably, geneset enrichment analysis revealed an upregulation in key autophagy‐related genesets: KEGG_lysosome (*P* = 3.52E‐05) and Reactome_Lysosome vesicle biogenesis (*P* = 0.0002) (Fig. 4*B*). Interestingly, there was also downregulation of numerous genesets involved in energy metabolism: Reactome_Respiratory electron transport (*P* = 3.29E‐12), KEGG_Oxidative phosphorylation (*P* = 5.70E‐09), and Reactome_Pyruvate metabolism and citric acid cycle TCA cycle (*P* = 3.48E‐07) (Fig. 4*C*). Additional pathway analysis revealed an attenuation of electron transport chain (ETC) (Fig. 5*A*) gene expression, which was matched by a reduction in expression of mitochondrial ETC subunit complexes I (VDR‐KD 53 ± 30% *vs*. Control 100 ± 43%, *P* = 0.055) and IV [VDR‐KD 68 ± 26% fold change (FC) *vs*. Control 100 ± 0.31%, *P* = 0.056, Fig. 5*B*]. Further analysis of transcription factor (TF) enrichment demonstrated a marked downregulation of muscle development TFs, including multiple myocyte enhancer factor‐2 (*Mef2*)‐related genesets (MEF2_02 *P* = 4.04E‐06, MEF2_03 *P* = 1.17E‐05) (Supplemental file 2), which is critical for the control of muscle differentiation.

### In vitro VDR‐KD reduces myoblast proliferation and terminal differentiation

**Figure 6:**
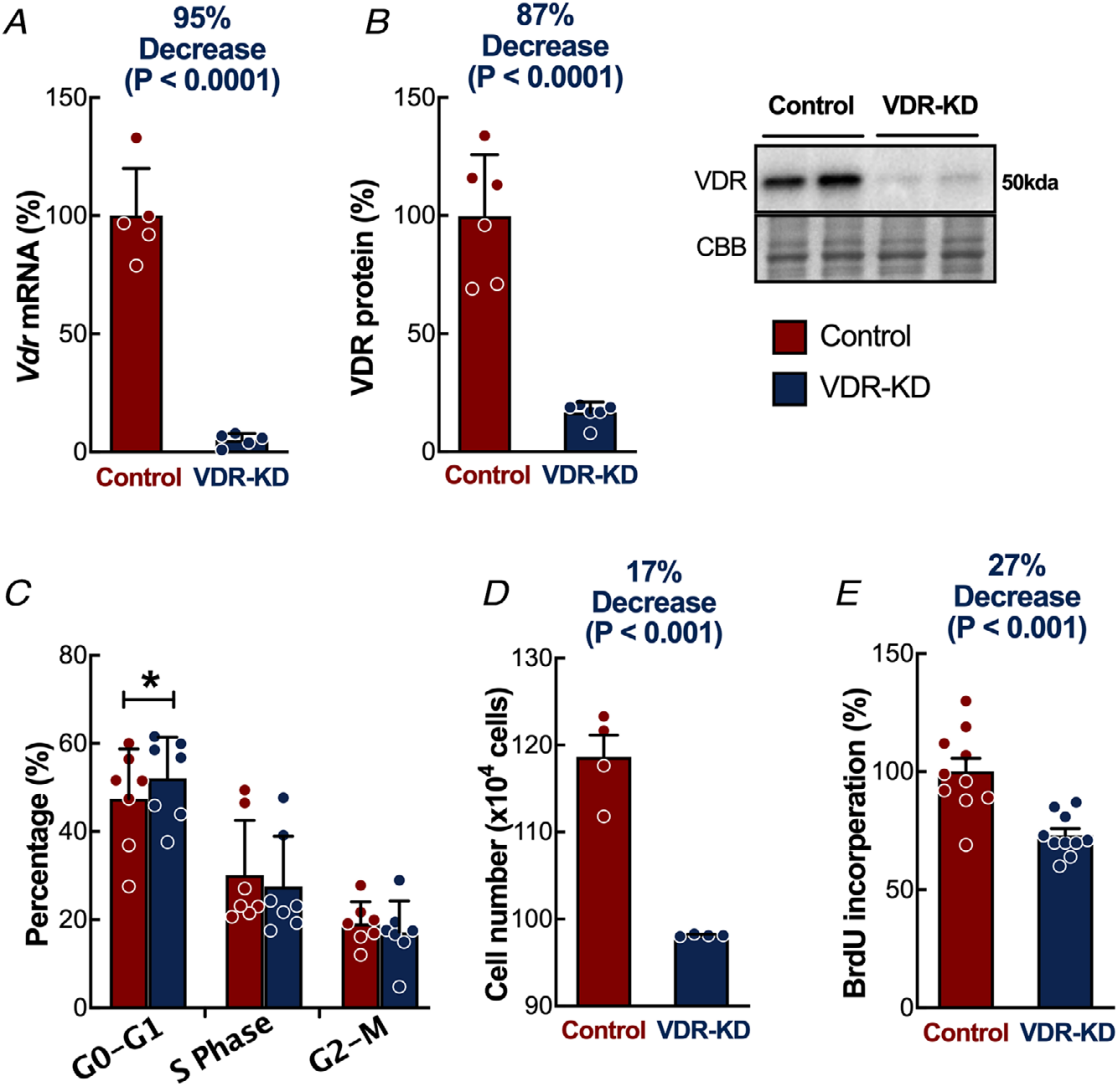
*In vitro* VDR‐KD impairs myoblast cell cycle regulation and proliferation *A*, qRT‐PCR analysis of VDR‐KD by shRNA (*n* = 5). *B*, representative western blot and quantification showing VDR‐KD (*n* = 6). *C*, cell cycle proportions of proliferating myoblasts (*n* = 7). *D*, total cell populations (*n* = 4). *E*, BrdU incorporation within proliferating myoblasts (*n* = 10). Data are individual values with mean ± SD, ^*^ *P* < 0.05. Data were analysed using *t*‐tests. [Color figure can be viewed at wileyonlinelibrary.com]

Given the large number of genes associated with myogenesis that were impacted by *in vivo* VDR‐KD, we next investigated whether VDR might have a pro‐myogenic role by generating C2C12 murine cells harbouring shRNA lentiviral VDR knockdown (*Vdr*, VDR‐KD −95 ± 3% *vs*. Control 100 ± 20%, *P* = 0.0001, Fig. 6*A* and *B*), with the hypothesis that VDR‐KD *in vitro* would impair myogenesis. Using flow cytometry and the fluorescent probe PI (for nuclear staining), we found that larger numbers of VDR‐KD myoblasts were in the G0–G1 phase of the cell cycle (VDR‐KD 52.1 ± 9.5% *vs*. Control 47.4 ± 11.4%, *P* = 0.038, Fig. 6*C*). Consistent with this, the total number of VDR‐KD myoblasts 96 h after seeding was lower (VDR‐KD 98 × 10^4^ ± 0.14 × 10^4^ cells *vs*. Control 118 × 10^4^ ± 5.1 × 10^4^ cells, *P* = 0.001, Fig. 6*D*), and this was associated with a reduction in DNA synthesis [BrdU incorporation VDR‐KD 73.1 ± 8.7% FC *vs*. Control 100 ± 17.5% FC, *P* = 0.0004, Fig. 6*E*) and thus attenuated proliferation.

**Figure 7:**
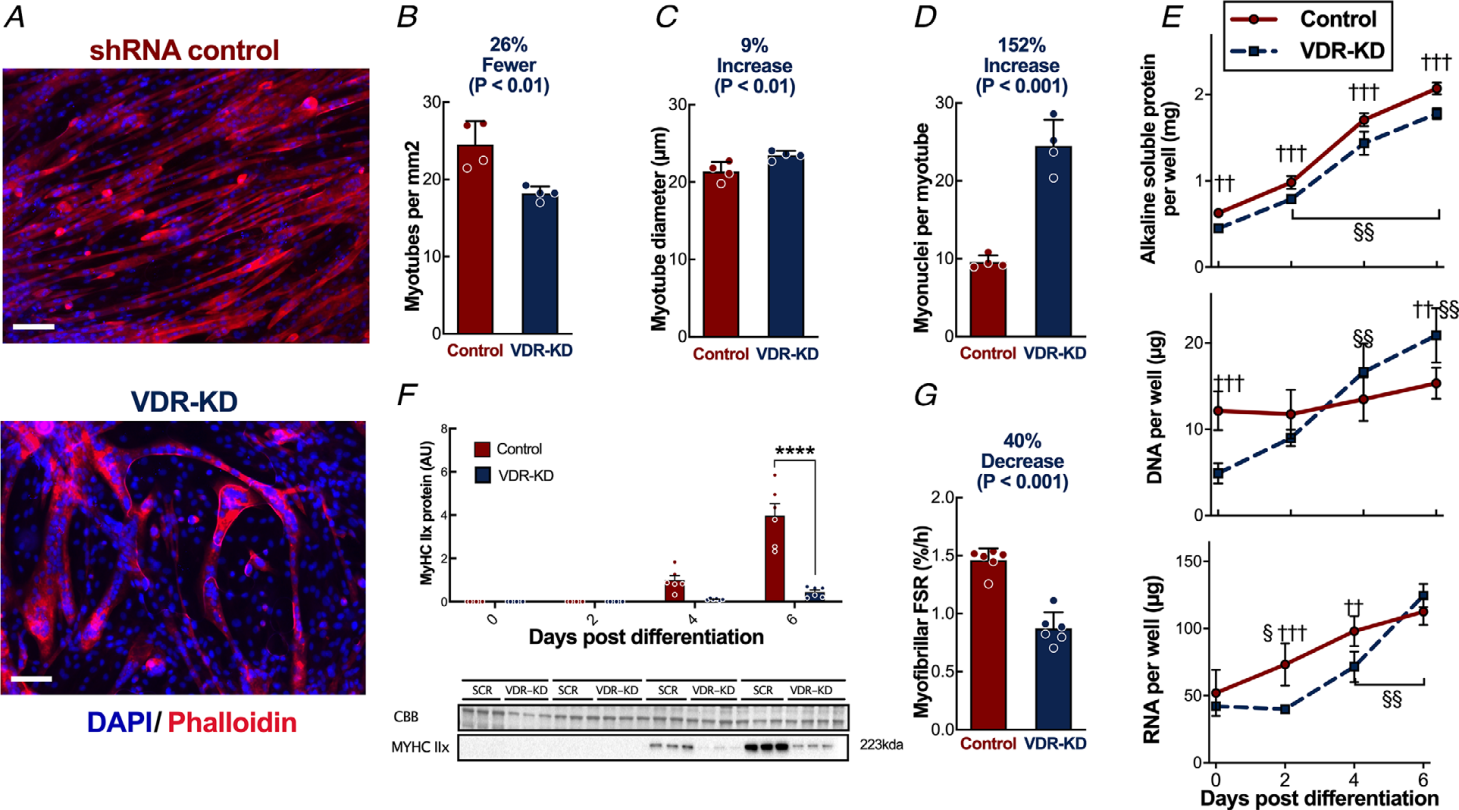
*In vitro* VDR‐KD impairs myogenesis and differentiation *A*, representative images of myotubes 5 days after differentiation, stained with phalloidin (red) and DAPI (blue). *B*, quantification of myotube population (*n* = 4). *C*, myotube diameter quantification (*n* = 4). *D*, quantification of the number of myonuclei per myotube (*n* = 4). *E*, total alkaline soluble protein, DNA and RNA content throughout differentiation (*n* = 6). F, western blot analysis of myosin IIx expression throughout differentiation (*n* = 6). *G*, myofibrillar protein synthesis rates 5 days after differentiation (*n* = 6). Scale bars represent 100 μm. For all grouped plots non‐pairwise comparisons were made using two‐sided *t*‐tests. For time‐course measurements, ANOVA with multiple comparison by Tukey analysis were used. Data are individual values with mean ± SD. ^*^ *P* < 0.05, ^****^ *P* < 0.0001 between the groups indicated. ^††^ *P* < 0.01, ^†††^ *P* < 0.001 between VDR‐KD and shRNA controls at that time point; ^§^ *P* < 0.05, ^§§^ *P* < 0.01 *versus* the initial time point. [Color figure can be viewed at wileyonlinelibrary.com]

Next, we studied the impact of VDR‐KD on terminal differentiation and observed fewer VDR‐KD myotubes than scramble‐infected myotubes (VDR‐KD 18.2 ± 1 myotube FC *vs*. Control 24.6 ± 3 myotube, *P* = 0.007, Fig. 7*A* and *B*). Surprisingly, the myotubes that did differentiate were larger (VDR‐KD 23.5 ± 0.6 μm *vs*. Control 21.4 ± 1.2 μm, *P* = 0.034, Fig. 7*C*). Consistent with an impairment of differentiation in VDR‐KD myotubes, we observed an increase in the number of myonuclei (VDR‐KD 24.5 ± 3.4 myonuclei *vs*. Control 9.6 ± 0.9 myonuclei, *P* = 0.0002, Fig. 7*D*), coupled with sustained increases in DNA content, despite serum depletion (DNA day 6, VDR‐KD 20.9 ± 3.2 μg *vs*. Control 15.4 ± 1.8 μg, *P* = 0.006, Fig. 7*E*). Moreover, protein and RNA content were lower in VDR‐KD myotubes on days 2 and 4 (RNA day 4, VDR‐KD 71.7 ± 11.5 μg *vs*. Control 98.1 ± 11.1 μg, *P* = 0, Fig. 7*E*). This was also reflected in a lack of myosin induction throughout the differentiation of VDR‐KD myotubes [Myosin day 6, VDR‐KD 0.45 ± 0.24 arbitrary units (AU) *vs*. Control 3.98 ± 1.37 AU, *P* < 0.0001, Fig. 7*F*]. Finally, myofibrillar MPS was lower in VDR‐KD cells than in scramble controls (VDR‐KD 0.87 ± 0.14% h *vs*. Control 1.46 ± 0.10% h, *P* < 0.0001, Fig. 7*G*). Together, these findings indicate that a reduction in VDR expression negatively influences myogenic processes by impairing myoblast proliferation and terminal differentiation.

## Discussion

Although recent work has demonstrated impaired regulation of skeletal muscle mass in the presence of vitamin D deficiency or low VDR expression (Dhanwal *et al*. 2013; Girgis *et al*. 2019), the function of the VDR in relation to skeletal muscle mass regulation remained poorly defined. Here, IVE transfection techniques were used to achieve short‐term but sustained muscle VDR‐KD in an internally controlled (i.e. contralateral limb) model, which also overcame the confounders of developmental dysregulation and dietary confounders (Amling *et al*. 1999; Endo *et al*. 2003). Using a combination of targeted and untargeted approaches, we show VDR loss‐of‐function rapidly induces muscle atrophy, and that this is specifically associated with an upregulation of autophagic processes.

First, we found a reduction of function in the VDR *in vivo* resulted in muscle atrophy at the level of individual fibres, along with an explanatory decrease in protein content. This is consistent with the findings of previous studies which showed that deletion of the VDR results in lower whole‐body lean mass and function (Girgis *et al*. 2019), which occurs secondary to myofibre atrophy (Endo *et al*. 2003; Girgis *et al*. 2015). Muscle atrophy may occur as a result of a reduction in MPS, an increase in MPB or a combination of the two, culminating in a negative net protein balance (Rudrappa *et al*. 2016). In the present study, no reductions in mRNA translation‐related signalling (i.e. AKT/mTORc1) were observed, nor were there detectable changes in mRNA translational efficiency/capacity processes (i.e. ribosomal content or directly quantified global/fraction‐specific rates of MPS; Brook *et al*. 2016). Collectively, these data demonstrate that while the vitamin D/VDR augments AKT/mTORc1 signalling (Salles *et al*. 2013), no loss occurs during acute VDR deficiency. Weaker anabolic signalling (via RPS6 and P70S6K) has been observed in response to vitamin D deficiency (18 weeks) (Gogulothu *et al*. 2020); however, this is probably due to systemic chronic responses to vitamin D deficiency. Interestingly, it was previously proposed that vitamin D deficiency induces muscle atrophy through greater proteasome‐mediated degradation (Bhat *et al*. 2013). However, we observed no modulation of proteasomal pathways; instead, previous findings may be related to Ca^2+^ dysregulation, as evidenced by the expression of proteasomal markers and partial rescue of atrophy by Ca^2+^ supplementation (Bhat *et al*. 2013).

In contrast, targeted measurement of proteolytic markers in VDR‐KD muscle indicated an induction of autophagosome formation (phosphorylated AMPK; Kim *et al*. 2011), in addition to an upregulation of lysosomal enzymes (at the mRNA and protein level). Crucially, there were increases in the expression of Beclin 1 and LC3B‐II, vital contributors to autophagosome formation, which demonstrates active autophagy induction (Wu *et al*. 2006). A role for the vitamin D‐VDR axis in the regulation of autophagy has been previously reported. Incubation of vitamin D with neurones, macrophages and MCF‐7 cells induces autophagy, while basal levels of autophagy are much higher in VDR‐KO MCF‐7 cells and mouse mammary glands (Yuk *et al*. 2009; Tavera‐Mendoza *et al*. 2017), which suggests that LC3B is constitutively repressed by the VDR (Tavera‐Mendoza *et al*. 2017). Vitamin D and subsequent VDR induction attenuates the dysfunction of autophagy that occurs in response to traumatic brain injury (Cui *et al*. 2017). Moreover, IL6/STAT3, which are essential regulators of autophagy, are upregulated in atrophied VDR‐KO muscle, and this is partially rescued through STAT3 inhibition (Gopinath, 2017). Recent investigations have suggested direct VDR induction of IL6 and STAT3 phosphorylation in dendritic cells (Català‐Moll *et al*. 2020), which further suggests a key role for the VDR in autophagy regulation. The present findings extend this to skeletal muscle and illustrate that *in vivo* ablation of the VDR may not actively induce autophagy; rather, its expression is inhibitory, preventing muscle protein degradation. Consistent with preliminary findings, follow‐up global RNA‐seq analysis demonstrated upregulation of established muscle atrophy‐related genesets (lysosomal, including vesicle biogenesis and NF‐kappaB signalling) (Jackman *et al*. 2013). Furthermore, VDR is a well‐established TF, and ChIP‐Seq datasets indicate that it has wide effects on gene expression (Satoh & Tabunoki, 2013) and regulates autophagy‐related genes. These data build on and help to explain previous observations that genetic loss of the VDR results in a reduction in fibre size (through poorly defined mechanisms; Endo *et al*. 2003; Girgis *et al*. 2015), and perhaps the clinical links between differences in VDR expression and muscle atrophy in disease (Bischoff‐Ferrari *et al*. 2004a; Punzi *et al*. 2012). Indeed, in agreement with our findings, it has been suggested that VDR‐KO‐mediated atrophy may occur through unexplained reductions in protein content, rather than necrosis (i.e. protein degradation, not apoptosis) (Endo *et al*. 2003).

Analysis of the RNA‐seq data revealed down‐regulation of numerous mitochondrial and energy metabolism‐related processes that have not previously been described in response to VDR‐KD. Consistent with this, TF analysis of the RNA‐seq data revealed that MEF2 TFs, which are essential for ATP production, complex I activity (She *et al*. 2011), and the maintenance of mitochondrial integrity and gene expression (e.g. PGC1‐α) (Naya *et al*. 2002), were markedly down‐regulated by VDR‐KD, which implies that VDR is required for normal mitochondrial function. The importance of VDR expression has previously been suggested in other cell types: its expression has been shown to be vital in keratinocytes, MCF‐7 cells and fibroblasts for normal mitochondrial regulation (Ricca *et al*. 2018), and there is some evidence that it may localize to the mitochondria (Silvagno *et al*. 2010). Indeed, cultured myoblasts exhibit lower mitochondrial respiration in response to VDR‐KD, potentially through impaired mitochondrial organization, membrane permeability or calcium handling (Ashcroft *et al*. 2020), which implies that VDR is required for normal mitochondrial function in skeletal muscle. Interestingly, vitamin D may have additional regulatory functions. Recent work (*Ryan et al*. 2016) has demonstrated that the incubation of skeletal muscle cells with vitamin D increases mitochondrial oxygen consumption and fusion/fission dynamics, with ∼2000 associated mRNA gene expression changes that are dependent upon the VDR. Furthermore, vitamin D appears to have a physiological role, because supplementation in humans reduces muscle phosphocreatine recovery time (Sinha *et al*. 2013), which is an established index of mitochondrial oxidative capacity (*Arnold et al*. 1984). Here, the downregulation of multiple mitochondrial/TCA cycle genesets by VDR‐KD reaffirms that the VDR is required for the maintenance of mitochondrial energetics (Ricca *et al*. 2018) and highlights it as a potential therapeutic target. It may also be that the maintenance of mitochondria is upstream of muscle mass preservation (Romanello & Sandri, 2016; Coen *et al*. 2019).

Given the putative role of the VDR in myogenesis, we investigated the potential mechanisms further by developing a sustained VDR‐KD system *in vitro*, to study the impacts on proliferation and differentiation in skeletal muscle cells, somewhat reflecting the processes involved in development, although our short‐term post‐natal *in vivo* approach was designed not to capture these. Vitamin D administration provokes G_0_–G_1_ cell cycle arrest, with induction of cyclin D3, p21 and p27, which are important regulators of cell cycle withdrawal (Irazoqui *et al*. 2014). Moreover, it has been demonstrated that they are induced by VDR‐dependent genomic mechanisms (Irazoqui *et al*. 2014), with p21 being a vitamin D‐target gene, containing a VDR promoter region binding site (Liu *et al*. 1996). Our findings extend and help to explain these observations, revealing the VDR is required for both cell cycle progression (G_0_–G_1_ transition) and transition to differentiation. Importantly, MPS in established muscles remain unchanged (i.e. *in vivo*), demonstrating the decreased *in vitro* MPS is due to lack of myosin induction, highlighting the VDR requirement for successful differentiation and development of muscle fibres. Indeed, this clarifies why muscle developmental dysregulation occurs in *in vivo* VDR‐KO models prior to study, which preclude the physiological characterization of a post‐natal role of the VDR (Endo *et al*. 2003; Girgis *et al*. 2019). This may be evidenced by the lack of overall differentiation in VDR‐KD cells (reflecting ongoing DNA synthesis) and the creation of very few, large myotubes. In contrast, mature skeletal muscle is post‐mitotic and has limited capacity for satellite cell DNA synthesis (Drake *et al*. 2015). Previous *in vitro* studies have demonstrated that exogenous vitamin D positively mediates myofibre hypertrophy (Salles *et al*. 2013) and increases myosin expression (Garcia *et al*. 2011; van der Meijden *et al*. 2016) in a VDR‐dependent fashion (Buitrago *et al*. 2013). Thus, concordant suppression of myofibrillar MPS and a lack of myosin induction in VDR‐KD myotubes may explain the reductions in muscle mass and fibre size in VDR‐null mice (Endo *et al*. 2003; Girgis *et al*. 2015), highlighting the key autonomous role of the VDR in myoblast differentiation, which does not require vitamin D stimulation.

Finally, it is important to note the limitations of this investigation. While these data demonstrate robust muscle fibre atrophy 10 days after IVE, earlier measures would allow for temporal CSA comparisons to be made. This would clarify whether VDR‐KD‐induced fibre atrophy was as a result of an arrest in fibre growth or overall reduction in CSA. Similarly, later measures (i.e. >10 days) may have permitted reductions in gene expression to manifest in protein expression. Furthermore, we did not directly measure the impact VDR‐KD would have upon muscle function (i.e. strength). Moreover, while anabolic signalling remained unchanged, loss of the VDR may impact stimulation of these pathways by exercise or diet. Future studies may be able to clarify such responses and whether a reduction in VDR expression (as seen in aged muscle) may impair muscle mass gains.

## Conclusion

The current study establishes an autonomous role of the VDR in skeletal muscle mass regulation, with lower expression eliciting myofibre atrophy through an induction of autophagy‐related pathways, and myogenic dysregulation. Furthermore, the VDR plays a fundamental modulatory role in skeletal muscle mitochondrial function. These data suggest that modulation of VDR expression or VDR‐targeting therapies may positively regulate skeletal muscle mass.

## Additional information

### Competing interests

No competing interests declared.

### Author contributions

J.J.B., K.S., N.J.S., M.E.C and P.J.A. designed the experiments. Cell lines were generated by A.A.K. J.J.B., D.A. and M.E.C. carried out *in vivo* sample collection; J.J.B., A.N., C.S.D., D.J.W., J.T. and F.K. performed data collection; J.J.B., M.S.B., D.J.W., B.E.P., A.P., J.T., F.K., K.S., I.J.G., N.J.S., M.E.C. and P.J.A. analysed the data. S.P.A. undertook mitochondrial protein complex measures. J.J.B. undertook RNA extraction for RNA‐seq, with I.J.G. performing bioinformatic analysis. J.J.B. and A.M.G. constructed the pathway analysis. J.J.B., M.S.B., D.J.W. and K.S. performed mass spectrometry analysis. All authors contributed to the preparation and drafting of the manuscript.

### Funding

MRC: Philip J Atherton, MR/J500495/1.

## Supporting information


**VDR‐KD**

**TF‐GSEA**

**Statistical Summary Document**

## Data Availability

GEO data are available in the repository (GSE110507). https://www.ncbi.nlm.nih.gov/geo/query/acc.cgi?acc=GSE110507.

## References

[tjp14489-bib-0001] Amling M , Priemel M , Holzmann T , Chapin K , Rueger JM , Baron R & Demay MB (1999). Rescue of the skeletal phenotype of vitamin D receptor‐ablated mice in the setting of normal mineral ion homeostasis: formal histomorphometric and biomechanical analyses. Endocrinology, 140, 4982–4987. 10537122 10.1210/endo.140.11.7110

[tjp14489-bib-0002] Andrews S. (2010). FastQC: a quality control tool for high throughput sequence data. Available online at: http://www.bioinformatics.babraham.ac.uk/projects/fastqc

[tjp14489-bib-0003] Arnold DL , Matthews PM & Radda GK (1984). Metabolic recovery after exercise and the assessment of mitochondrial function in vivo in human skeletal muscle by means of 31P NMR. Magn Reson Med 1, 307–315. 6571561 10.1002/mrm.1910010303

[tjp14489-bib-0004] Arthur W. Ham MDL ( 1934 ) . Hypervitaminosis D rickets: the action of vitamin D. Br J Exp Pathol 15, 228. Available at: http://www.ncbi.nlm.nih.gov/pmc/articles/PMC2065028/ [Accessed 20 April 2016].

[tjp14489-bib-0005] Ashcroft SP , Bass JJ , Kazi AA , Atherton PJ & Philp A (2020). The vitamin D receptor regulates mitochondrial function in C2C12 myoblasts. Am J Physiol Cell Physiol 318, C536–C541. 31940245 10.1152/ajpcell.00568.2019 PMC7099523

[tjp14489-bib-0006] Bass JJ , Wilkinson DJ , Rankin D , Phillips BE , Szewczyk NJ , Smith K & Atherton PJ (2017). An overview of technical considerations for Western blotting applications to physiological research. Scand J Med Sci Sport 27, 4–25. 10.1111/sms.12702 PMC5138151 27263489

[tjp14489-bib-0007] Bhan A , Rao AD & Rao DS (2010). Osteomalacia as a result of vitamin D deficiency. Endocrinol Metab Clin North Am 39, 321–331, table of contents. 20511054 10.1016/j.ecl.2010.02.001

[tjp14489-bib-0008] Bhat M , Kalam R , Syh Qadri S , Madabushi S & Ismail A (2013). Vitamin D deficiency‐induced muscle wasting occurs through the ubiquitin proteasome pathway and is partially corrected by calcium in male rats. Endocrinology 154, 4018–4029. 23928374 10.1210/en.2013-1369

[tjp14489-bib-0009] Bischoff‐Ferrari HA , Borchers M , Gudat F , Dürmüller U , Stähelin HB & Dick W (2004a). Vitamin D receptor expression in human muscle tissue decreases with age. J Bone Miner Res 19, 265–269. 14969396 10.1359/jbmr.2004.19.2.265

[tjp14489-bib-0010] Bischoff‐Ferrari HA , Dietrich T , Orav EJ , Hu FB , Zhang Y , Karlson EW & Dawson‐Hughes B (2004b). Higher 25‐hydroxyvitamin D concentrations are associated with better lower‐extremity function in both active and inactive persons aged >or = 60 y. Am J Clin Nutr 80, 752–758. 15321818 10.1093/ajcn/80.3.752

[tjp14489-bib-0011] Bolger AM , Lohse M & Usadel B (2014). Trimmomatic: a flexible trimmer for Illumina sequence data. Bioinformatics 30, 2114–2120. 24695404 10.1093/bioinformatics/btu170 PMC4103590

[tjp14489-bib-0012] Brook MS , Wilkinson DJ , Mitchell WK , Lund JN , Phillips BE , Szewczyk NJ , Greenhaff PL , Smith K & Atherton PJ (2016). Synchronous deficits in cumulative muscle protein synthesis and ribosomal biogenesis underlie age‐related anabolic resistance to exercise in humans. J Physiol 594, 7399–7417. 27654940 10.1113/JP272857 PMC5157077

[tjp14489-bib-0013] Buitrago C , Pardo VG & Boland R (2013). Role of VDR in 1α,25‐dihydroxyvitamin D3‐dependent non‐genomic activation of MAPKs, Src and Akt in skeletal muscle cells. J Steroid Biochem Mol Biol 136, 125–130. 23470620 10.1016/j.jsbmb.2013.02.013

[tjp14489-bib-0014] Català‐Moll F , Li T , Ciudad L , Rodríguez‐Ubreva J & Ballestar E (2020). Vitamin D receptor and STAT3 cooperate to establish TET2‐mediated tolerogenesis. bioRxiv 2020.02.28.969634.

[tjp14489-bib-0015] Ceglia L , Niramitmahapanya S , da Silva Morais M , Rivas DA , Harris SS , Bischoff‐Ferrari H , Fielding RA & Dawson‐Hughes B (2013). A randomized study on the effect of vitamin D_3_ supplementation on skeletal muscle morphology and vitamin D receptor concentration in older women. J Clin Endocrinol Metab 98, E1927–1935. 24108316 10.1210/jc.2013-2820 PMC3849671

[tjp14489-bib-0016] Cleasby ME , Davey JR , Reinten TA , Graham MW , James DE , Kraegen EW & Cooney GJ (2005). Acute bidirectional manipulation of muscle glucose uptake by in vivo electrotransfer of constructs targeting glucose transporter genes. Diabetes 54, 2702–2711. 16123360 10.2337/diabetes.54.9.2702

[tjp14489-bib-0017] Cleasby ME , Reinten TA , Cooney GJ , James DE & Kraegen EW (2007). Functional studies of Akt isoform specificity in skeletal muscle in vivo; maintained insulin sensitivity despite reduced insulin receptor substrate‐1 expression. Mol Endocrinol 21, 215–228. 17021050 10.1210/me.2006-0154

[tjp14489-bib-0018] Close GL , Russell J , Cobley JN , Owens DJ , Wilson G , Gregson W , Fraser WD & Morton JP (2013). Assessment of vitamin D concentration in non‐supplemented professional athletes and healthy adults during the winter months in the UK: implications for skeletal muscle function. J Sports Sci 31, 344–353. 23083379 10.1080/02640414.2012.733822

[tjp14489-bib-0019] Coen PM , Musci RV , Hinkley JM & Miller BF (2019). Mitochondria as a target for mitigating sarcopenia. Front Physiol, 10.3389/fphys.2018.01883. PMC6335344 30687111

[tjp14489-bib-0020] Crossland H , Kazi A A, Lang CH , Timmons J A, Pierre P , Wilkinson DJ , Smith K , Szewczyk NJ & Atherton PJ (2013). Focal adhesion kinase is required for IGF‐I‐mediated growth of skeletal muscle cells via a TSC2/mTOR/S6K1‐associated pathway. Am J Physiol Endocrinol Metab 305, E183–93. 23695213 10.1152/ajpendo.00541.2012 PMC3725543

[tjp14489-bib-0021] Cui C , Cui J , Jin F , Cui Y , Li R , Jiang X , Tian Y , Wang K , Jiang P & Gao J (2017). Induction of the vitamin D receptor attenuates autophagy dysfunction‐mediated cell death following traumatic brain injury. Cell Physiol Biochem 42, 1888–1896. 28772270 10.1159/000479571

[tjp14489-bib-0022] Dev R , Del Fabbro E , Schwartz GG , Hui D , Palla SL , Gutierrez N & Bruera E (2011). Preliminary report: vitamin D deficiency in advanced cancer patients with symptoms of fatigue or anorexia. Oncologist 16, 1637–1641. 21964001 10.1634/theoncologist.2011-0151 PMC3233299

[tjp14489-bib-0023] Dhanwal DK , Dharmshaktu P , Gautam VK , Gupta N & Saxena A (2013). Hand grip strength and its correlation with vitamin D in Indian patients with hip fracture. Arch Osteoporos 8, 158. 24146354 10.1007/s11657-013-0158-8

[tjp14489-bib-0024] Drake JC , Bruns DR , Peelor FF , Biela LM , Miller RA , Miller BF & Hamilton KL (2015). Long‐lived Snell dwarf mice display increased proteostatic mechanisms that are not dependent on decreased mTORC1 activity. Aging Cell 14, 474–482. 25720574 10.1111/acel.12329 PMC4406676

[tjp14489-bib-0025] Endo I , Inoue D , Mitsui T , Umaki Y , Akaike M , Yoshizawa T , Kato S & Matsumoto T (2003). Deletion of vitamin D receptor gene in mice results in abnormal skeletal muscle development with deregulated expression of myoregulatory transcription factors. Endocrinology 144, 5138–5144. 12959989 10.1210/en.2003-0502

[tjp14489-bib-0026] Forrest KYZ & Stuhldreher WL (2011). Prevalence and correlates of vitamin D deficiency in US adults. Nutr Res 31, 48–54. 21310306 10.1016/j.nutres.2010.12.001

[tjp14489-bib-0027] Garcia L a, King KK , Ferrini MG , Norris KC & Artaza JN (2011). 1,25(OH)_2_vitamin D3 stimulates myogenic differentiation by inhibiting cell proliferation and modulating the expression of promyogenic growth factors and myostatin in C2C12 skeletal muscle cells. Endocrinology 152, 2976–2986. 21673099 10.1210/en.2011-0159 PMC3138228

[tjp14489-bib-0028] Gasier HG , Fluckey JD & Previs SF (2010). The application of 2H_2_O to measure skeletal muscle protein synthesis. Nutr Metab 7, 31. 10.1186/1743-7075-7-31 PMC2873296 20409307

[tjp14489-bib-0029] Girgis CM , Cha KM , Houweling PJ , Rao R , Mokbel N , Lin M , Clifton‐Bligh RJ & Gunton JE (2015). Vitamin D receptor ablation and vitamin D deficiency result in reduced grip strength, altered muscle fibers, and increased myostatin in mice. Calcif Tissue Int 97, 602–610. 26340892 10.1007/s00223-015-0054-x

[tjp14489-bib-0030] Girgis CM , Cha KM , So B , Tsang M , Chen J , Houweling PJ , Schindeler A , Stokes R , Swarbrick MM , Evesson FJ , Cooper ST & Gunton JE (2019). Mice with myocyte deletion of vitamin D receptor have sarcopenia and impaired muscle function. J Cachexia Sarcopenia Muscle 10, 1228–1240. 31225722 10.1002/jcsm.12460 PMC6903451

[tjp14489-bib-0031] Girgis CM , Clifton‐Bligh RJ , Mokbel N , Cheng K & Gunton JE (2014a). Vitamin D signaling regulates proliferation, differentiation, and myotube size in C2C12 skeletal muscle cells. Endocrinology 155, 347–357. 24280059 10.1210/en.2013-1205

[tjp14489-bib-0032] Girgis CM , Mokbel N , Cha KM , Houweling PJ , Abboud M , Fraser DR , Mason RS , Clifton‐Bligh RJ & Gunton JE (2014b). The vitamin D receptor (VDR) is expressed in skeletal muscle of male mice and modulates 25‐hydroxyvitamin D (25OHD) uptake in myofibers. Endocrinology 155, 3227–3237. 24949660 10.1210/en.2014-1016 PMC4207908

[tjp14489-bib-0033] Gogulothu R , Nagar D , Gopalakrishnan S , Garlapati VR , Kallamadi PR & Ismail A (2020). Disrupted expression of genes essential for skeletal muscle fibre integrity and energy metabolism in vitamin D deficient rats. J Steroid Biochem Mol Biol 197, 105525. 31705962 10.1016/j.jsbmb.2019.105525

[tjp14489-bib-0034] Gopinath SD (2017). Inhibition of Stat3 signaling ameliorates atrophy of the soleus muscles in mice lacking the vitamin D receptor. Skelet Muscle 7, 2. 28122601 10.1186/s13395-017-0121-2 PMC5264327

[tjp14489-bib-0035] Halfon M , Phan O & Theta D (2015). Vitamin D: A review on its effects on muscle strength, the risk of fall, and frailty. Biomed Res Int, 10.1155/2015/953241. PMC4427016 26000306

[tjp14489-bib-0036] van Iersel MP , Kelder T , Pico AR , Hanspers K , Coort S , Conklin BR & Evelo C (2008). Presenting and exploring biological pathways with PathVisio. BMC Bioinformatics 9, 399. 18817533 10.1186/1471-2105-9-399 PMC2569944

[tjp14489-bib-0037] Irazoqui AP , Boland RL & Buitrago CG (2014). Actions of 1,25(OH)_2_‐vitamin D3 on the cellular cycle depend on VDR and p38 MAPK in skeletal muscle cells. J Mol Endocrinol 53, 331–343. 25316911 10.1530/JME-14-0102

[tjp14489-bib-0038] Jackman RW , Cornwell EW , Wu C‐L & Kandarian SC (2013). Nuclear factor‐κB signalling and transcriptional regulation in skeletal muscle atrophy. Exp Physiol 98, 19–24. 22848079 10.1113/expphysiol.2011.063321 PMC3505235

[tjp14489-bib-0039] Kim J , Kundu M , Viollet B & Guan K‐L (2011). AMPK and mTOR regulate autophagy through direct phosphorylation of Ulk1. Nat Cell Biol 13, 132–141. 21258367 10.1038/ncb2152 PMC3987946

[tjp14489-bib-0040] Kutmon M , van Iersel MP , Bohler A , Kelder T , Nunes N , Pico AR & Evelo CT (2015). PathVisio 3: an extendable pathway analysis toolbox. PLoS Comput Biol 11, 1–13. 10.1371/journal.pcbi.1004085 PMC4338111 25706687

[tjp14489-bib-0041] Kutmon M , Riutta A , Nunes N , Hanspers K , Willighagen EL , Bohler A , Mélius J , Waagmeester A , Sinha SR , Miller R , Coort SL , Cirillo E , Smeets B , Evelo CT & Pico AR (2016). WikiPathways: capturing the full diversity of pathway knowledge. Nucleic Acids Res 44, D488–D494. 26481357 10.1093/nar/gkv1024 PMC4702772

[tjp14489-bib-0042] Liao Y , Smyth GK & Shi W (2013). The Subread aligner: fast, accurate and scalable read mapping by seed‐and‐vote. Nucleic Acids Res 41, e108. 23558742 10.1093/nar/gkt214 PMC3664803

[tjp14489-bib-0043] Liberzon A , Subramanian A , Pinchback R , Thorvaldsdóttir H , Tamayo P & Mesirov JP (2011). Molecular signatures database (MSigDB) 3.0. Bioinformatics 27, 1739–1740. 21546393 10.1093/bioinformatics/btr260 PMC3106198

[tjp14489-bib-0044] Liu M , Lee MH , Cohen M , Bommakanti M & Freedman LP (1996). Transcriptional activation of the Cdk inhibitor p21 by vitamin D3 leads to the induced differentiation of the myelomonocytic cell line U937. Genes Dev 10, 142–153. 8566748 10.1101/gad.10.2.142

[tjp14489-bib-0045] Lun ATL , Chen Y & Smyth GK (2016). It's DE‐licious: A recipe for differential expression analyses of RNA‐seq experiments using quasi‐likelihood methods in edgeR. Methods Mol Biol 1418, 391–416. 27008025 10.1007/978-1-4939-3578-9_19

[tjp14489-bib-0046] Scimeca M , Centofanti F , Celi M , Gasbarra E , Novelli G , Botta A & Tarantino U (2018). Vitamin D receptor in muscle atrophy of elderly patients: a key element of osteoporosis‐sarcopenia connection. Aging Dis 9, 952–964. 30574409 10.14336/AD.2018.0215 PMC6284754

[tjp14489-bib-0047] van der Meijden K , Bravenboer N , Dirks NF , Heijboer AC , den Heijer M , de Wit GMJ , Offringa C , Lips P & Jaspers RT (2016). Effects of 1,25(OH)2D3 and 25(OH)D3 on C2C12 myoblast proliferation, differentiation, and myotube hypertrophy. J Cell Physiol 231, 2517–2528. 27018098 10.1002/jcp.25388 PMC5111790

[tjp14489-bib-0048] Morgan M , Falcon S & Gentleman R (2017). GSEABase: Gene set enrichment data structures and methods.

[tjp14489-bib-0049] Naya FJ , Black BL , Wu H , Bassel‐Duby R , Richardson JA , Hill JA & Olson EN (2002). Mitochondrial deficiency and cardiac sudden death in mice lacking the MEF2A transcription factor. Nat Med 8, 1303–1309. 12379849 10.1038/nm789

[tjp14489-bib-0050] Norman AW (2008). From vitamin D to hormone D: fundamentals of the vitamin D endocrine system essential for good health. Am J Clin Nutr 88, 491S–499S. 18689389 10.1093/ajcn/88.2.491S

[tjp14489-bib-0051] Okuno H , Kishimoto KN , Hatori M & Itoi E (2012). 1α,25‐dihydroxyvitamin D_3_ enhances fast‐myosin heavy chain expression in differentiated C2C12 myoblasts. Cell Biol Int 36, 441–447. 22276695 10.1042/CBI20100782

[tjp14489-bib-0052] Pike JW (2014). Expression of the vitamin D receptor in skeletal muscle: are we there yet? Endocrinology 155, 3214–3218. 25152176 10.1210/en.2014-1624 PMC4138570

[tjp14489-bib-0053] Pittas AG , Lau J , Hu FB & Dawson‐Hughes B (2007). The role of vitamin D and calcium in type 2 diabetes. A systematic review and meta‐analysis. J Clin Endocrinol Metab 92, 2017–2029. 17389701 10.1210/jc.2007-0298 PMC2085234

[tjp14489-bib-0054] Prabhala A , Garg R & Dandona P (2000). Severe myopathy associated with vitamin D deficiency in western New York. Arch Intern Med 160, 1199–1203. 10789615 10.1001/archinte.160.8.1199

[tjp14489-bib-0055] Punzi T , Fabris A , Morucci G , Biagioni P , Gulisano M , Ruggiero M & Pacini S (2012). C‐reactive protein levels and vitamin d receptor polymorphisms as markers in predicting cachectic syndrome in cancer patients. Mol Diagn Ther 16, 115–124. 22497530 10.1007/BF03256436

[tjp14489-bib-0056] Ricca C , Aillon A , Bergandi L , Alotto D , Castagnoli C & Silvagno F (2018). Vitamin D receptor is necessary for mitochondrial function and cell health. Int J Mol Sci 19, 1672. 10.3390/ijms19061672 PMC6032156 29874855

[tjp14489-bib-0057] Rizzoli R , Boonen S , Brandi ML , Burlet N , Delmas P & Reginster JY (2008). The role of calcium and vitamin D in the management of osteoporosis. Bone 42, 246–249. 18055288 10.1016/j.bone.2007.10.005

[tjp14489-bib-0058] Robinson MD , McCarthy DJ & Smyth GK (2010). edgeR: a Bioconductor package for differential expression analysis of digital gene expression data. Bioinformatics 26, 139–140. 19910308 10.1093/bioinformatics/btp616 PMC2796818

[tjp14489-bib-0059] Robinson MD & Oshlack A (2010). A scaling normalization method for differential expression analysis of RNA‐seq data. Genome Biol 11, R25. 20196867 10.1186/gb-2010-11-3-r25 PMC2864565

[tjp14489-bib-0060] Romanello V & Sandri M (2016). Mitochondrial quality control and muscle mass maintenance. Front Physiol 6, 1–21. 10.3389/fphys.2015.00422 PMC4709858 26793123

[tjp14489-bib-0061] Rudrappa SS , Wilkinson DJ , Greenhaff PL , Smith K , Idris I & Atherton PJ (2016). Human skeletal muscle disuse atrophy: effects on muscle protein synthesis, breakdown, and insulin resistance—a qualitative review. Front Physiol 7, 361. 27610086 10.3389/fphys.2016.00361 PMC4997013

[tjp14489-bib-0062] Ryan ZC , Craig TA , Folmes CD , Wang X , Lanza IR , Schaible NS , Salisbury JL , Nair KS , Terzic A , Sieck GC & Kumar R (2016). 1α,25‐Dihydroxyvitamin D_3_ regulates mitochondrial oxygen consumption and dynamics in human skeletal muscle cells. J Biol Chem 291, 1514–1528. 26601949 10.1074/jbc.M115.684399 PMC4714233

[tjp14489-bib-0063] Salles J , Chanet A , Giraudet C , Patrac V , Pierre P , Jourdan M , Luiking YC , Verlaan S , Migné C , Boirie Y & Walrand S (2013). 1,25(OH)_2_‐vitamin D3 enhances the stimulating effect of leucine and insulin on protein synthesis rate through Akt/PKB and mTOR mediated pathways in murine C2C12 skeletal myotubes. Mol Nutr Food Res 57, 2137–2146. 23929734 10.1002/mnfr.201300074

[tjp14489-bib-0064] Sandri M (2013). Protein breakdown in muscle wasting: role of autophagy‐lysosome and ubiquitin‐proteasome. Int J Biochem Cell Biol 45, 2121–2129. 23665154 10.1016/j.biocel.2013.04.023 PMC3775123

[tjp14489-bib-0065] Sato Y , Iwamoto J , Kanoko T & Satoh K (2005). Low‐dose vitamin D prevents muscular atrophy and reduces falls and hip fractures in women after stroke: a randomized controlled trial. Cerebrovasc Dis 20, 187–192. 16088114 10.1159/000087203

[tjp14489-bib-0066] Satoh J & Tabunoki H (2013). Molecular network of chromatin immunoprecipitation followed by deep sequencing‐based vitamin D receptor target genes. Mult Scler 19, 1035–1045. 23401126 10.1177/1352458512471873

[tjp14489-bib-0067] Schroeder A , Mueller O , Stocker S , Salowsky R , Leiber M , Gassmann M , Lightfoot S , Menzel W , Granzow M & Ragg T (2006). The RIN: an RNA integrity number for assigning integrity values to RNA measurements. BMC Mol Biol 7, 3. 16448564 10.1186/1471-2199-7-3 PMC1413964

[tjp14489-bib-0068] She H , Yang Q , Shepherd K , Smith Y , Miller G , Testa C & Mao Z (2011). Direct regulation of complex I by mitochondrial MEF2D is disrupted in a mouse model of Parkinson disease and in human patients. J Clin Invest 121, 930–940. 21393861 10.1172/JCI43871 PMC3049386

[tjp14489-bib-0069] Silvagno F , De Vivo E , Attanasio A , Gallo V , Mazzucco G & Pescarmona G (2010). Mitochondrial localization of vitamin D receptor in human platelets and differentiated megakaryocytes. PLoS One 5, e8670. 20107497 10.1371/journal.pone.0008670 PMC2809087

[tjp14489-bib-0070] Sinha A , Hollingsworth KG , Ball S & Cheetham T (2013). Improving the vitamin D status of vitamin D deficient adults is associated with improved mitochondrial oxidative function in skeletal muscle. J Clin Endocrinol Metab 98, E509–513. 23393184 10.1210/jc.2012-3592

[tjp14489-bib-0071] Srikuea R , Zhang X , Park‐Sarge O‐K & Esser KA (2012). VDR and CYP27B1 are expressed in C2C12 cells and regenerating skeletal muscle: potential role in suppression of myoblast proliferation. Am J Physiol Cell Physiol 303, C396–405. 22648952 10.1152/ajpcell.00014.2012 PMC3422988

[tjp14489-bib-0072] Tanaka M , Kishimoto KN , Okuno H , Saito H & Itoi E (2014). Vitamin D receptor gene silencing effects on differentiation of myogenic cell lines. Muscle Nerve 49, 700–708. 23873355 10.1002/mus.23950

[tjp14489-bib-0073] Tavera‐Mendoza LE , Westerling T , Libby E , Marusyk A , Cato L , Cassani R , Cameron LA , Ficarro SB , Marto JA , Klawitter J & Brown M (2017). Vitamin D receptor regulates autophagy in the normal mammary gland and in luminal breast cancer cells. Proc Natl Acad Sci U S A 114, E2186–E2194. 28242709 10.1073/pnas.1615015114 PMC5358377

[tjp14489-bib-0074] Wilkinson DJ , Franchi M V , Brook MS , Narici M V , Williams JP , Mitchell WK , Szewczyk NJ , Greenhaff PL , Atherton PJ & Smith K (2014). A validation of the application of D_2_O stable isotope tracer techniques for monitoring day‐to‐day changes in muscle protein subfraction synthesis in humans. Am J Physiol Endocrinol Metab 306, E571–579. 24381002 10.1152/ajpendo.00650.2013 PMC3948971

[tjp14489-bib-0075] Wu J , Dang Y , Su W , Liu C , Ma H , Shan Y , Pei Y , Wan B , Guo J & Yu L (2006). Molecular cloning and characterization of rat LC3A and LC3B–two novel markers of autophagosome. Biochem Biophys Res Commun 339, 437–442. 16300744 10.1016/j.bbrc.2005.10.211

[tjp14489-bib-0076] Wyon MA , Wolman R , Nevill AM , Cloak R , Metsios GS , Gould D , Ingham A & Koutedakis Y (2016). Acute effects of vitamin D3 supplementation on muscle strength in Judoka athletes: a randomized placebo‐controlled, double‐blind trial. Clin J Sport Med 26, 279–284. 26535872 10.1097/JSM.0000000000000264

[tjp14489-bib-0077] Yang D , Beylot M , Brunengraber DZ , Samols MA , Anderson VE & Brunengraber H (1998). Assay of low deuterium enrichment of water by isotopic exchange with [U‐13C3] acetone and gas chromatography ± mass spectrometry. Anal Biochem 258, 315–321. 9570847 10.1006/abio.1998.2632

[tjp14489-bib-0078] Yuk J‐M , Shin D‐M , Lee H‐M , Yang C‐S , Jin HS , Kim K‐K , Lee Z‐W , Lee S‐H , Kim J‐M & Jo E‐K (2009). Vitamin D3 induces autophagy in human monocytes/macrophages via cathelicidin. Cell Host Microbe 6, 231–243. 19748465 10.1016/j.chom.2009.08.004

